# Flame synthesis of carbon nanostructures on Ni-plated hardmetal substrates

**DOI:** 10.1186/1556-276X-6-331

**Published:** 2011-04-13

**Authors:** Hongmei Zhu, Tongchun Kuang, Bin Zhu, Shumei Lei, Zongwen Liu, Simon Peter Ringer

**Affiliations:** 1 School of Materials Science and Engineering, South China University of Technology, Guangzhou 510640, China; 2 Analytical and Testing Center, South China University of Technology, Guangzhou 510640, China; 3 Australian Centre for Microscopy and Microanalysis, The University of Sydney, Sydney, NSW 2006, Australia

## Abstract

In this article, we demonstrate that carbon nanostructures could be synthesized on the Ni-plated YG6 (WC-6 wt% Co) hardmetal substrate by a simple ethanol diffusion flame method. The morphologies and microstructures of the Ni-plated layer and the carbon nanostructures were examined by various techniques including scanning electron microscopy, X-ray diffraction, and Raman spectroscopy. The growth mechanism of such carbon nanostructures is discussed. This work may provide a strategy to improve the performance of hardmetal products and thus to widen their potential applications.

## Background

Hardmetals are widely used for cutting tools and as wear resistant components [1]. In order to improve the performance and the durability of the hardmetals, the application of coating is necessary. Carbon nanostructures such as nanocrystalline diamond, carbon nanofibers (CNFs), and carbon nanotubes (CNTs) are considered as the ideal coating or reinforcing candidates for the hardmetals due to their extraordinary mechanical, chemical, thermal, and electronic properties [2-4]. However, a poor adhesion of the coating on the hardmetal substrate is still the main limitation to the wide applications.

Many efforts have been made to improve the adhesion between the coating and the hardmetal substrate, the introduction of an interlayer was demonstrated as one of the most effective approaches to achieve this [5,6]. Here, we chose metal Ni as an interlayer considering its several inherent advantages [7-10]: (1) the linear expansion coefficient of Ni is very close to that of the hardmetal substrate; (2) Ni possesses favorable wettability with carbon nanostructures and thus catalyzes their nucleation and growth; and (3) Ni is hardly influenced by the temperature in combustion flame due to its outstanding heat resistance.

In comparison to other traditional methods, flames can naturally provide a source of both reactive hydrocarbon gas and elevated temperatures for large-scale synthesis of carbon nanostructures at higher energy utilization rate and at lower cost [7-13]. For example, ethanol diffusion flame has been reported to synthesize CNTs and CNFs on carbon steels, low alloy steels, Ni-containing metals such as type 304 and YUS701 austenitic stainless steels, and pure copper [8,12,13].

In this work, we demonstrate that carbon nanostructures could be successfully deposited on the Ni-plated YG6 hardmetal substrates by a simple ethanol diffusion flame method. The characterization of the as-prepared carbon nanostructures was carried out using scanning electron microscope (SEM), X-ray diffraction (XRD), and Raman spectroscopy. The effects of flame-deposition time and the metal catalyst on the growth of these carbon nanostructures were also investigated.

## Experimental

### Materials

The commercial YG6 hardmetal cutting tool inserts (WC-6 wt% Co, made by Zhuzhou cemented carbide corporation, Zhuzhou China style C116) were used as substrates. The electroplating bath composition includes nickel sulfate (NiSO_4_) 250 to 300 g/l, nickel chloride (NiCl_2_) 50 to 60 g/l, boric acid (H_3_BO_3_) 40 to 50 g/l, and some additives. The pH value of the electrolyte varied between 3.8 and 4.5, and the average *J*_k _(i.e., cathodic current density) was maintained at 2 A/dm^2^.

### Substrate process

An Ni layer of approximately 20 μm in thickness was electro-deposited on the hardmetal YG6 surface, and the electroplating process was performed in the following sequence: mechanical grinding (on the diamond discs of 240# → 400# → 600# → 800#, respectively) → chemical deoiling and degreasing → electro cleaning → acid pickling → alkali cleaning → nickel plating.

The electroplating process was carried out for 40 min at temperature of 50 to 65°C.

### Flame

**Figure 1:**
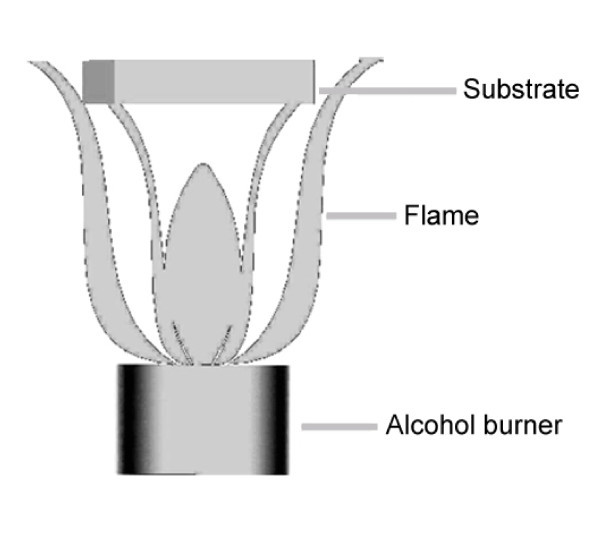
**A schematic representation of the experimental flame used in this work**.

**Figure 2:**
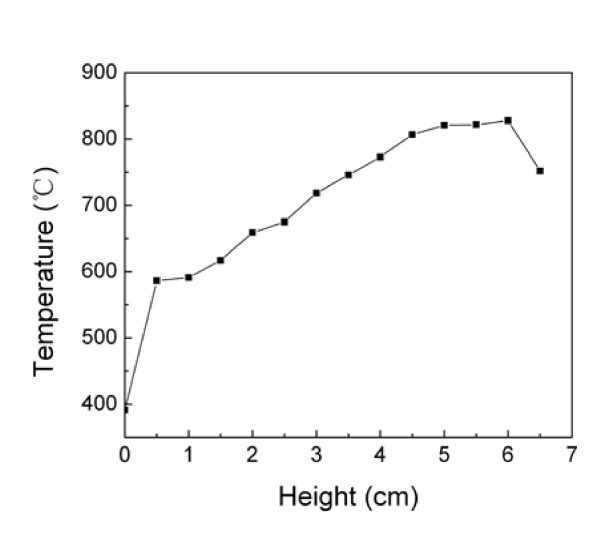
**The flame temperature distribution at different heights of the alcohol burner**. The abscissa denotes the vertical height relative to the flame nozzle and the ordinate denotes the corresponding temperature of the symmetrical core of the flame.

The diffusion flame was produced by a common laboratory alcohol burner using pure ethanol as the fuel. It is well known that a natural flame of the alcohol burner contains three distinct regions, namely the outer flame, the inner flame, and the flame center. However, only the inner flame of the incomplete combustion is plentifully dissociated with carbon particles, which are responsible for the growth of carbon materials [8,12,13]. In this study, the visible inner flame zone was located between 4 and 6 cm above the fuel nozzle of the alcohol burner. Considering the factors such as the flame size, temperature, and the unavoidable flame instability in the combustion process, the Ni-plated hardmetal substrates were inserted in the flame at 5 cm above the fuel nozzle. To make it clear, Figure 1 shows the position of the substrates in the flame, and the flame temperature distribution at different heights of the alcohol burner was shown in Figure 2. The flame temperature at 5 cm was measured about 800°C by a K-type thermocouple with a diameter of 1 mm. The synthesis time was set to 30 and 60 min. Hereafter, the hardmetal samples exposed to different time were referred to the 30-min sample and the 60-min sample, respectively.

### Characterization

Various techniques were used to characterize the carbon nanostructures grown on the surface of the hard metal. The particle characteristics (shape, size, and distribution) were examined by a Philip-XL30 FEG SEM. The composition and bonding information of the coating layer were obtained by XRD, recorded using a Rigaku D/max-IIIA (30 kV, 30 mA, Cu Kα) at a scanning rate of 0.5°/min in the 2θ range of 25 to 125°. Raman spectroscopy (Renishaw 2000, Ar laser wavelength 514 nm, 20 mW) was utilized to identify and analyze the microstructures of the coating.

## Results and discussion

The thickness of the interlayer is very crucial to achieve a better adhesion between the coating layer and the substrate. If the interlayer is too thin, cobalt (Co) element contained in the substrate could re-diffuse from inside to outside at high flame temperature, thus reducing the effect of the interlayer. If the interlayer is too thick, the performance of the hardmetal tools could be degraded since the Ni interlayer possesses lower hardness and heat resistance than the hardmetal substrate. Therefore, the thickness of the interlayer is generally set ranging from several to several tens micrometers [5,6].

The thickness of the electro-plated layer can be calculated by formula (1) according to the electro-chemical theory:(1)

where *δ *is the thickness of electro-plated layer (μm); *C *the electrochemical equivalent (g Ah^-1^), 1.095 for Ni; *t *the electrodeposition time; *η*_k _the current efficiency, here 85%; *ρ *the density of metal-plated layer (g cm^-3^), 8.908 for Ni. Therefore, the thickness of the Ni-plated layer is amounted to 20 μm.

**Figure 3:**
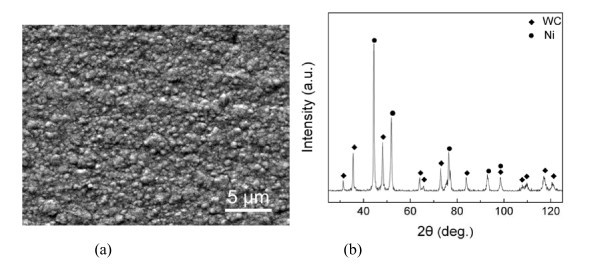
**The SEM morphology and XRD pattern of the Ni-plated layer deposited on the YG6 hardmetal substrate**. (a) SEM morphology and (b) XRD pattern.

Figure 3 shows the SEM morphology and XRD pattern of the as-prepared Ni-plated layer on the YG6 hardmetal substrate. The Ni-plated layer is extremely bright, smooth, and compact, and no peeling and cracking were visible. The SEM result shows that the Ni particles are slightly inhomogeneous in sizes and orientations (Figure 3a). The large particles are 0.5 to 1.0 μm and the small ones are 50 to 100 nm in diameter. The XRD pattern (Figure 3b) reveals that the major composites were the matrix WC and metal Ni, and no other impurities were detected in the Ni-plated layer. Moreover, the sharp peaks suggest that both the components of WC and Ni crystallized very well in the reported conditions. According to the Debye-Scherra formula, the calculated Ni particle size is around 0.5 μm, which is in good agreement with the SEM observation (Figure 3a).

**Figure 4:**
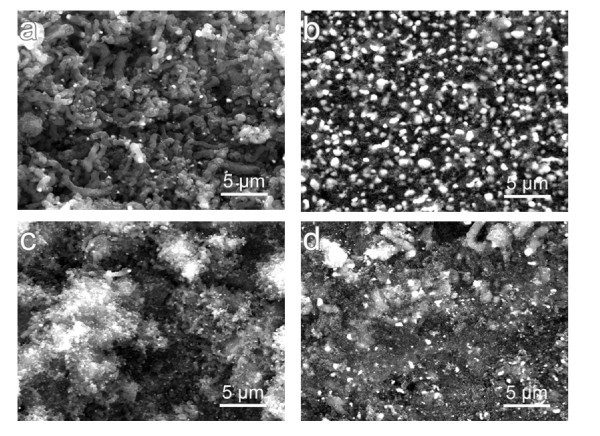
**The SEM morphologies of the carbon nanostructures deposited in different zones for different time lengths**. The *upper images *are the center zone (a) and the marginal zone (b) of the 30-min sample, and the *lower images *are the center zone (c) and the marginal zone (d) of the 60-min sample.

Figure 4 shows the SEM images of the carbon nanostructures deposited on the YG6 hardmetal with the Ni-plated interlayer. Due to the limited flame size, different contact zones would form on the hardmetal substrate. After a 30-min deposition, the flame-deposited materials in the center zone (corresponding to the inner flame) can be seen in Figure 4a. A close inspection showed that the nanofibrous carbon materials (i.e., CNFs/CNTs) grew disorderly and entangled with each other. The carbonaceous sizes are in a wide distribution and the longest ones are 3 to 4 μm in length. This can be attributed to two possible reasons. One is the slightly inhomogeneous electro-plated Ni particles and the other is the relatively weaker catalytic activity in the low-temperature marginal zone than that in the high-temperature center zone. In contrast, the products on the marginal surface of the 30-min sample (corresponding to the outer flame) are generally uniform and continuous in flocculent shape as shown in Figure 4b. Apparently, it can be seen from the SEM images that the tips of the carbon nanostructures are attached by some small particles with light contrast. The composition of these embedded nanoparticles was verified to metal Ni by energy dispersive X-ray spectroscopy (EDS, not shown here). As reported previously [7-10], the Ni nanoparticles acted as a catalyst for the formation of these carbon nanostructures.

When the time was extended to 60 min, the flame-deposited products display tube/wire-like morphology in both the center zone and the marginal zone (Figure 4c, d). In this study, it was found that the yield of the nanofibrous carbon materials increased with extending deposition time. According to Choi et al. [14], the carbon atoms existed in form of both CNTs and carbon nanoparticles in the initial stage, and the hydrogen atoms produced during incomplete combustion of ethanol began to etch the flame-deposited material after the generation of carbon nanoparticles. Consequently, CNTs gradually dominated the product as time progressing due to the higher stability of CNTs than that of carbon nanoparticles. It should be noted that it is difficult to detect the internal structure of the as-deposited carbon nanostructures within the resolution of SEM. A further examination by transmission electron microscopy (TEM) is needed to confirm whether the flame product is CNFs or CNTs or the combination of both.

**Figure 5:**
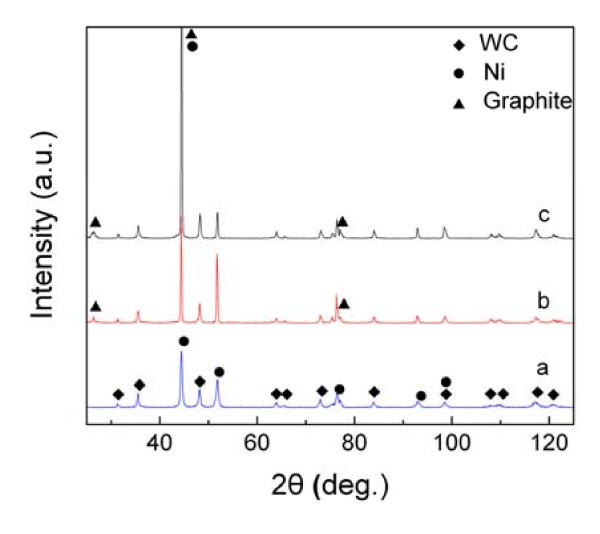
**The XRD patterns of the Ni-plated substrate and the flame-deposited products for different time lengths**. (a) the Ni-plated substrate, (b) the 30-min sample and (c) the 60-min sample.

Figure 5 provides the XRD patterns of the original Ni-plated YG6 hardmetal substrate and the flame-deposited carbon nanostructures. The reflections occurred at around 26.4°, 44.5°, and 77.0° can be attributed to graphite, which correspond to the crystal planes of (002), (101), and (110), respectively, according to the International Centre for Diffraction Data (ICDD, #65-6212)

By comparison of the three spectrums in Figure 5, it can be concluded that the highest peak at 44.5° is composed of both Ni and graphite. The 60-min sample shows higher intensity peaks of graphite than those of the 30-min sample, which is closely associated with a lager amount of carbonaceous material caused by the prolonged flame-deposition time. This is very consistent with the SEM observation as seen in Figure 4.

**Figure 6:**
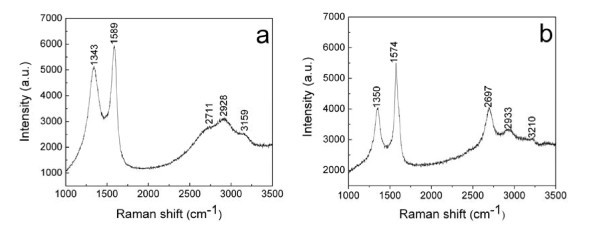
**The Raman spectra of the flame-deposited carbon nanostructures for different time lengths**. (a) the 30-min sample and (b) the 60-min sample.

Raman spectroscopy is a sensitive, convenient, and non-destructive technique for characterizing the microstructure of the carbon nanostructures [15]. Figure 6 shows the Raman spectra of the as-deposited carbon nanostructures after different flame-deposition time lengths. The peak assignments of the Raman spectra are summarized in Table 1. It is well known that the two peaks located at approximately 1350 and 1580 cm^-1 ^are called as D-band and G-band of graphite, respectively. The D band is attributed to the disorder-induced vibration of C-C bond, and the G band corresponds to the C-C vibration of the carbon material with a *sp*^2 ^orbital structure. Therefore, the relative band intensity (*I*_D_/*I*_G_) is related to the graphitic structure of the combustion material [15]. As clearly seen from Table 1, the value of *I*_D_/*I*_G _is 0.86 and 0.73 for the 30-min sample and the 60-min sample, respectively. This indicates that the as-deposited carbon nanostructures here possess a relatively higher degree of order and graphitization. Moreover, the graphitization degree of the 60-min sample is obviously higher than that of the 30-min sample, which is in good agreement with the analytic results by SEM (Figure 4) and XRD (Figure 5).

**Table 1:** The Raman spectroscopic parameters for the flame-deposited carbon nanostructures.

Sample	D mode (cm^-1^)	G mode (cm^-1^)	D* mode (cm^-1^)	(D + G) mode(cm^-1^)	2G mode (cm^-1^)	*I*_D_/*I*_G_
The 30-min sample	1343	1589	2711	2928	3159	0.86
The 60-min sample	1350	1574	2697	2933	3210	0.73

Various growth mechanisms for carbon nanostructures, including nanocrystalline diamond, CNFs, and CNTs, have been proposed [16]. In our present work, the existence of Ni particles at the tip of the nanofibrous carbon material shows good evidence of the metal-catalyzed growth [7-10]. It is well documented that Ni is capable of catalyzing the nucleation and growth of CNFs/CNTs due to a weak affinity for carbon [7-10]. However, different crystal planes of Ni exhibit different preferences for the epitaxial matches with carbon as well as different activities for the decomposition of the hydrocarbons [17]. Based on the existing models for carbon nanomaterial synthesis [7-14,16,17], the growth process of the carbon nanostructures deposited on Ni-plated hardmetal substrates in the current ethanol diffusion flames could be divided into three stages. First, the fuel ethanol pyrolysed into abundant carbonaceous radical species such as C_2_, C_3_, C_4_, and CO, which precipitated on the active crystal planes of the catalytic Ni particles as mentioned above. Secondly, the pyrolytic hydrocarbons and carbon clusters deposited on the surface of the catalytic Ni particles, meanwhile the hydrocarbon products continued to decompose into other smaller carbon-containing substances. Thirdly, catalyzed by Ni particles, these carbon precursors diffused from one active crystal plane of the catalytic Ni particles to another and finally deposited in the form of CNFs/CNTs on the hardmetal substrate.

## Conclusions

We have demonstrated in this work that the ethanol diffusion flame method could be used to synthesize carbon nanostructures on Ni-plated YG6 hardmetal substrates. The quality and the graphitization degree of the flame-deposited carbon nanostructures were significantly enhanced with the increase of deposition time. The characteristics (grain size, shape, and distribution) of the Ni catalyst had a crucial influence on the growth of the carbon nanostructures. In addition, due to the unsteady flame and carbon supply during combustion, inhomogeneous carbon nanostructures were fabricated eventually. These findings could provide a new insight for enhancing the performance of hardmetals by a simple flame method.

## Abbreviations

CNFs: carbon nanofibers; CNTs: carbon nanotubes; EDS: energy dispersive X-ray spectroscopy;, ICDD: International Centre for Diffration Data; SEM: scanning electron microscope; TEM: transmission electron microscopy; XRD: X-ray diffraction.

## Competing interests

The authors declare that they have no competing interests.

## Authors' contributions

HZ conducted all the experiments and drafted the manuscript.

TK designed the experiments and supervised the whole study.

BZ participated in measurements and data analysis.

SL helped in experiments and characterization.

ZL and SPR provided the financial and technical support to the study.

All the authors read and approved the final manuscript.
